# Start from Scratch: A Crowdsourcing-Based Data Fusion Approach to Support Location-Aware Applications †

**DOI:** 10.3390/s19204518

**Published:** 2019-10-17

**Authors:** Yonghang Jiang, Bingyi Liu, Ze Wang, Xiaoquan Yi

**Affiliations:** 1 Department of Computer Science, City University of Hong Kong, Hong Kong; yhjiang4-c@my.cityu.edu.hk; 2 School of Computer Science and Technology, Wuhan University of Technology, Wuhan 430070, China; cs_wangze@whut.edu.cn (Z.W.); yixiaoquan@whut.edu.cn (X.Y.)

**Keywords:** internet of things, crowdsourcing, indoor localization, data fusion

## Abstract

As one of the most important breakthroughs for modern transportation, the indoor location-based technology has been gradually penetrating into our daily lives and underlines the foundation of the Internet of Things (IoT). To improve the positioning accuracy and efficiency, crowdsourcing has been widely applied in indoor localization in recent years. However, the crowdsourced data can hardly be fused easily to enable usable applications for the reason that the data are collected by different users, in different locations, at different times, with different noises and distortions. Although different data fusing methods have been implemented in different crowdsourcing services, we find that they may not fully leverage the data collected from multiple dimensions that can potentially lead to a better fusion results. In order to address this problem, we propose a more general solution, which can fuse the multi-dimensional crowdsourced data together and align them with the consistent time and location stamps, by using the features of the sensory data only, and thus build high quality crowdsourcing services from the raw data samplings collected from the environment. Finally, we conduct extensive evaluations and experiments using different commercial devices to validate the effectiveness of the method we proposed.

## 1. Introduction

With the proliferation of wireless communication and Internet of Things (IoT), location-based services have shaped and enabled a wide range of applications for our safety and convenience. While GPS dominates outdoor localization and becomes an essential element of the modern transportation, the prospect of indoor navigation has also gained attention in the last few decades as it holds important value for ubiquitous applications in location-based service (LBS) such as inventory management, logistical and supply chain management, smart home and smart building monitoring, retails and sport analytics, mall navigation, virtual reality, etc.

However, indoor location is inaccessible for GPS signals due to structural blockages and severe multiple propagation effects. Currently, there is no general solution for indoor localization, but there are several potential technologies available to provide indoor positioning and map construction solutions such as radio frequency identification (RFID), ultra wide band (UWB), micro-electromechanical systems (MEMS) multi-sensors, and wireless local area networks (WLAN). To fully leverage the data collected from multiple dimensions that can potentially lead to a better indoor positioning result, crowdsourcing has drawn significant attention in recent years. More and more crowdsourcing approaches have been presented, and the popularity of smart devices boosts their applications. The term crowdsourcing describes a new computing paradigm that distributes the work previously handled by employees to a large undefined network of individuals. Obviously, a crowd of people can coordinate and solve problems faster than a single person, and the crowd can generate data rapidly about the particular location.

Such model is an efficient way for indoor location-aware applications. The crowdsourced data, however, can hardly be fused easily to enable usable applications for the reason that the data are collected by different users, in different locations, at different times, with different noises and distortions. At first, the sensory readings are inaccurate due to the deviation of low-cost sensing devices, and different individuals can report slightly inconsistent readings on the same parameter. Since the crowdsourced data possess the advantage of their large quantity, many researchers propose to improve the accuracy through data fusion. These methods, however, may suffer from the fact that crowdsourced data traces are distorted both temporally and spatially. As a result, in this case, the fusion of multiple data sources can hardly be proceeded. The lack of precise time and location information largely limits the usability of the crowdsourcing data, and thus there is an urgent need for efficient crowdsourced data alignment to support varying applications.

As the time calibration can be achieved through time synchronization, we focus on the geographical trace alignment in this paper. Theoretically, collecting data from different locations should be based on a localization service. However, in the indoor scenarios, the indoor positioning systems are not widely deployed and used in practice. There are many existing indoor localization approaches such as the fingerprint-based methods [1,2,3,4,5], the ranging based methods [6], and the like. They, however, may suffer from the high dynamics and complexities of the indoor environment and thus output erroneous results. The authors in [7,8] propose the methods to combine the sensor information with the constraints imposed by the map, thereby filtering out infeasible locations and converging on the true location. However, they need special constraints of the floor plan, which may not always be available, as the floor plan cannot be acquired in some circumstances. A recent state-of-the-art approach Walkie-Markie [9] presents an indoor pathway mapping scheme. It automatically reconstructs the internal pathway maps of buildings, using the trend of WiFi signals as landmarks to calibrate the location of pathways. This method, however, we find still has room to be further improved due to several limitations. Firstly, as this system works based on WiFi-Marks, it is less capable of processing short data traces with hardly any WiFi-Marks. Moreover, it assumes that the distortions of traces are simple because they only come from incorrect stride lengths and headings. As a matter of fact, due to a variety of localization methods, the distortions could be more complicated in practice. Some other works use geomagnetic information [10,11] or WiFi information [12,13] for indoor localization. Although a high localization performance can be achieved, they focus on the feature of a single data dimension (such as WiFi), i.e., they cannot be applied to fuse other sensory data with different features from WiFi signals. Moreover, in the indoor environment, there are various signals and noises which will interfere with the feature of the single data dimension, and thus may affect their positioning accuracy.

**Figure 1:**
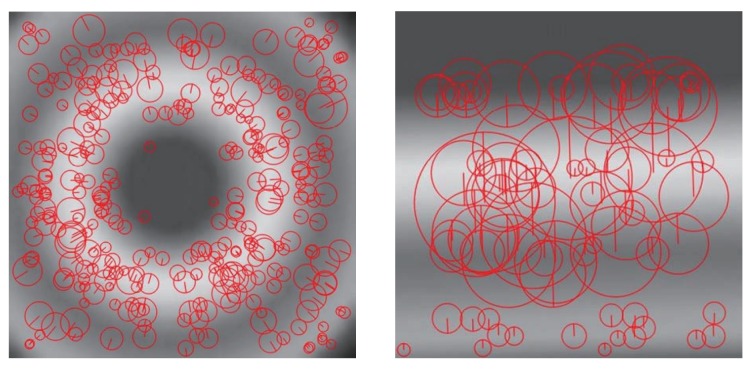
Gradient of AP signal and magnetic field in ideal models.

In order to address this problem, we propose a novel solution that aligns the multi-dimensional crowdsourced data with the right time and location stamps, and thus builds high quality traces from raw data samplings. Generally, crowdsourced traces consist of multiple data dimensions such as the WiFi signal strength, the ambient temperature/humidity, the magnetic field information, and the like. Each dimension corresponds to one underlying parameter of the physical world and exhibits its unique feature on the data distribution and variation. For example, the wireless signal follows the strength loss model and the magnetic follows the magnetic field model. This field feature seams consistent as the fluctuations of gradient along a specific trace, even if the deviations varies for different devices. Figure 1 shows the gradient of access point (AP) and magnetic field in ideal models. More importantly, with only one dimension information, the trace may not be unique, e.g., the trace departing from the same AP to different directions can have a similar decrease gradient feature. However, the multi-dimensional information, e.g., signals from more than one AP and base stations, three directional components of magnetic field, etc, could give us the chance to conduct integrated fusion and align traces accurately without a priori knowledge of the environment.

Based on these observations, we form the crowdsourced trace alignment as a multi-dimensional data consistency optimization problem and search for the more accurate solution based on the mutual correlation among the multi-dimensional data. In summary, the contributions of this work are as follows:

- We extensively study the problem of multi-dimensional crowdsourced data fusion problem and propose a novel method to fuse noisy and distorted crowdsourced data in different environments.
- We propose a new trace coding and transformation approach to conduct the trace alignment, in order to deal with a complex trace distortion issue.
- We propose an optimization scheme to find the crowdsourced trace alignment solution.
- We conduct extensive evaluations and experiments to validate the effectiveness of our method.

The rest of this paper is organized as follows. In Section 2, we discuss existing efforts related to this work. Section 3 provides a detailed statement of the crowdsourcing data alignment problem. Section 4 describes our main idea and the framework of the proposed approach. In Section 5, we discuss the 3D indoor trace collection and illustrate the evaluation results. We reach a conclusion in Section 6.

## 2. Related Work

There are some existing efforts on map reconstruction that are related to our work. FootSLAM [14] proposes a Bayesian estimation approach that applies foot mounted inertial sensors to construct the internal map. PlaceSLAM [15] incorporates human-reported place information. Escort [16] explores the possibility of using mobile phone sensors and opportunistic user-intersections to navigate users. UnLoc [17] uses environmental signatures as landmarks to re-calibrate users’ locations, which is an unsupervised localization approach to avoid the labor intensive war-driving for building the localization feature dataset. In this paper, we further explore the distribution of multi-dimensional data and conduct integrated optimization on all dimensions for the data alignment task, where the primary version of the paper is published in [18]. A recent state-of-the-art approach Walkie-Markie [9] is an indoor pathway mapping system. It automatically reconstructs internal pathway maps of buildings without any a priori knowledge, using the trend of WiFi signals as landmarks to calibrate the location of pathways. Walkie-Markie, however, considers only one dimension of the sensory data and focuses on pathways that can hardly be applied to an open indoor space.

Moreover, there are some existing approaches on indoor localization [2,6,8] and navigation [10,12,19,20] that are related to this work. A major category of indoor localization approaches is fingerprint-based, which collects measurements at known locations in advance. The fingerprint collection process is usually called a site survey. In the localization stage, the fingerprint-based methods compare the observed measurements with the fingerprints in the database and determine the new locations according to the best matched fingerprints. Many different types of measurements can be applied as fingerprints, such as radio-frequency (RF) [1], GSM [2], FM radio signal [4], geo-magnetism [3], CIR (channel impulse response) [5], and the like. [11] puts forward a kind of fingerprint distinguishability evaluation model to find a high discernible fingerprint extracted method for indoor localization, e.g., the authors in [13] propose a novel deep learning framework for indoor localization tasks using WiFi fingerprints. In these works, the need for a survey is a key bottleneck since it is labor-intensive and time-consuming. Especially in large-scale applications, fingerprints that store the buildings’ environment characteristics must be mapped. As collecting fingerprints in thousands of buildings is quite costly, some researchers propose crowdsourcing approaches [21,22] to conduct a site survey. The authors in [23] provide an unsupervised learning-based location labeling technique for crowdsourced fingerprints, then generate indoor high-precision maps, and provide indoor localization and navigation services.

With the rich on-board sensors on mobile devices, the mobile crowdsourcing (MCS) [24,25] appears through mixing the mobile techniques and crowdsourcing, which can enable many location-aware crowdsourced tasks. Recently, the MCS sensing applications have provided benefits to many aspects of people’s everyday lives. For example, TrafficInfo [26] can provide a real-time public transport information service through sharing information and sending feedback with participatory sensing. Ear-Phone [27] can generate a noise map to facilitate monitoring of environmental urban noise pollution through crowdsourcing data collection. In addition, MCS sensing applications can provide a solution to the lack of floor plans of indoor localization service. The authors in [28,29,30] propose the methods for reconstructing the indoor floor plans by utilizing inertial sensors (accelerometers, compasses and gyroscopes), visual and spatial information crowdsourced from the users’ smart phones. Since such applications rely on users’ participation, many incentive mechanisms [31,32] have been proposed to encourage users to share their sensed data. At the same time, when participating in crowdsourcing tasks, users may suffer security and privacy issues. The authors in [33,34] have studied the privacy leaks and potential threats in crowdsourcing tasks to handle such issues. Moreover, the collected data in crowdsourcing tasks are usually not well structured and noisy. Thus, some data analysis technologies are proposed to improve the quality of collected data. For instance, [35] proposes a quality-sensitive answering model to satisfy user required accuracy through guiding the crowdsourcing query engine to process and monitor human tasks that can provide an estimated accuracy for each generated result. The authors in [36] propose an anchor point-based forward-backward smoothing method to generate indoor localization databases and a quantitative framework to evaluate the quality of sensed data automatically. Conversely, there are some works that motivate users to provide high-quality data through data quality control [37,38].

Although the aforementioned methods are important for improving the positioning accuracy and supporting location-aware applications, few of which consider the solution of multi-dimensional crowdsourced trace fusion and alignment to deal with complex trace distortion issues. Moreover, the realistic indoor scenario, such as the coexistence of multi-dimensional data, has not been fully considered in the literature. Motivated by these facets, we propose a framework to find the crowdsourced trace alignment solution in this paper.

## 3. Problem Statements and Definitions

In common crowdsourcing applications, the collected data can be represented as an ordered set of trituples {(d,t,p)}. For each tuple, *d* denotes the sensory data, which may contain multiple dimensions such as wireless signal strength, magnetic field, acoustic information, etc.; *t* is the time stamp and *p* is the location stamp.

Given stamps *t* and *p*, the ground truth of *d* is unknown in most cases due to the existence of measurement errors and noises. Nevertheless, a large amount of noisy samplings may be collected by a crowd of *N* mobile devices, say $\left\{P_{j}\right\}$. In this work, we denote the crowdsourced data as $d+n_{j}$ in which $n_{j}$ is the additive noise part. Consequently, we will discuss the properties of the crowdsourced data sets.

### 3.1. Distortion of Crowdsourced Data

For the three tuples of one record, the time stamp is relatively easy for alignment through the time synchronization. The distortion of the collected data from crowd mainly results from two sources: the volumetric aspect and geometric aspect. The inaccurate or uncalibrated on-broad sensors can report noisy readings and the geometric distortion is caused by the inaccurate localization. Firstly, we consider the noise in sensory values. Let (x,y,z) denote the coordinates of a location in which *z* indicates the floor number. Different from the outdoor environment, buildings can have multiple floors and the data traces are collected on different floors. To the real value d(x,y,z) of a physical parameter, the observed measurement could be:

(1) $$\begin{array}{c} o(x,y,z)=d(x,y,z)+n(x,y,z). \end{array}$$

In order to derive the real value d(x,y,z) from o(x,y,z), many researchers propose to use the fusion techniques. For example, some approaches assume that the noise n(x,y,z) comes from different devices are independent. As a result, n(x,y,z) can be regarded as a random variable following the zero-mean Gaussian distribution. In this case, averaging *N* different noisy measurements from different traces can achieve a good estimation of the real value,

$$\begin{array}{c} \bar{o}\left(x,y,z\right)=\frac{1}{N}\sum_{j=1}^{N}o_{j}\left(x,y,z\right). \end{array}$$

Then, it follows that

$$\begin{array}{c} E\{\bar{o}\left(x,y,z\right)\}=d\left(x,y,z\right), \end{array}$$

where $E\{\bar{o}\left(x,y,z\right)\}$ is the expected value of $\bar{o}\left(x,y,z\right)$. Let $\sigma_{\bar{o}\left(x,y,z\right)}^{2}$ and $\sigma_{n(x,y,z)}^{2}$ denote the variances of $\bar{o}\left(x,y,z\right)$ and n(x,y,z), respectively. Then, we have:

$$\begin{array}{c} \sigma_{\bar{o}\left(x,y,z\right)}=\frac{1}{\sqrt{N}}\sigma_{n(x,y,z)}. \end{array}$$

As *N* increases, the equation above implies that the variability of the measurement at each location decreases. In typical crowdsourcing scenarios, when there are more than dozens of users, $\bar{o}\left(x,y,z\right)$ can achieve a good estimation about the noiseless value d(x,y,z) and the approximation accuracy increases as the user number increases.

In practice, the observations $\left\{o_{j}\left(x,y,z\right)\right\}$ should be aligned to the accurate location coordinates; otherwise, the averaging process does not provide a better fusion result. For the outdoor environment with GPS services, the average-based data fusion can be applied for a wild range of crowdsourced services. In the indoor environment, however, the lack of efficient localization services becomes the main obstruction for the data alignment and indoor map construction.

Employing an indoor localization method, the result comes out with an error for each location l=(x,y,z):

$$\begin{array}{c} p=l+e\left(x,y,z\right)=\left(x+e_{x},y+e_{y},z+e_{z}\right). \end{array}$$

May existing localization methods are fingerprint based, whose performance degrades if the sensory data are inaccurate and unstable. For example, in a localization approach using AP signal strength as the fingerprint, the change of the furniture layout or AP position can significantly affect the signal strength observed in the same location. In addition, different places can exhibit similar signal strength observations, leading to the sensing ambiguity.

### 3.2. Multi-Dimensional Data Alignment

In this work, we address the multi-dimensional data alignment problem that assigns proper locations to each data point by analyzing the multi-dimensional sensing traces.

Formally, each type of information is referred to as a *dimension* $m_{i}$, e.g., the signal strength of a specific AP or the geomagnetic field. Each observed trace $O_{j}$ is a set $\left\{\left(o_{j},t_{j},p_{j}\right)\right\}$ ordered by $t_{j}$. With multiple on-board sensors on the mobile device, the observed value $o_{j}$ contains information of multiple dimensions, e.g., $o_{j}=\left\{o_{j}^{i}\right\}$. Data in varying dimensions could possess different distributions. For example, the signal strength of a specific AP may exhibit a radial distribution centered at the AP’s location, and the geomagnetic field is approximately along the longitudes. Based on the observation, our proposed scheme tries to find an alignment of the data traces that best fits the underlying data distributions cross all the data dimensions.

## 4. Framework

### 4.1. Mechanism Overview

**Figure 2:**
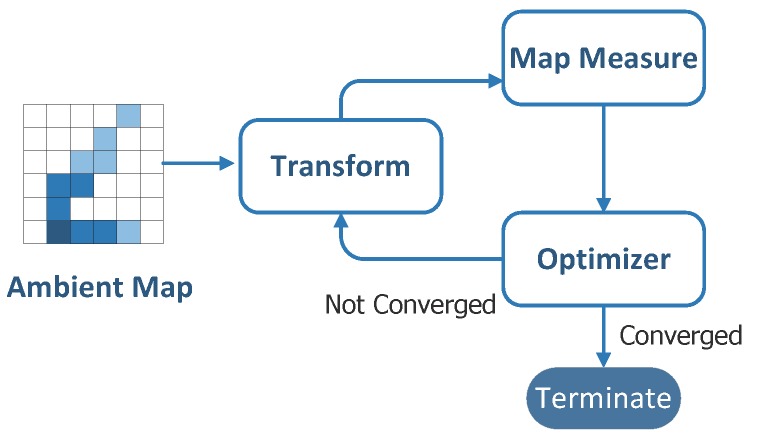
Mechanism overview.

As illustrated in Figure 2, our approach is an iterative method that basically consists of four coupled components: (1) a coding scheme that models the locations of a data trace; (2) a map metric that measures the quality of ambient trace data; (3) a class of transformations that can be applied to the traces during the fusion process; and (4) a solver that searches for the next transformation to improve the map-level data quality in all data dimensions.

For the purpose of measuring the effectiveness of the data alignment, we propose a map-level trace quality metric, $Q(M_{i})$. We denote $M_{i}$ as an *ambient map* of the $i^{th}$ data dimension. An *ambient map* is defined as the data map of a specific dimension. For example, the ambient map of Wi-Fi signal consists of all the signal strength readings at different physical locations. For the purpose of data fusion, the data map is usually embedded into grids, and data samplings within the same cell are fused together. In our analysis, we propose to use multi-resolution ambient maps for the reason that different data dimensions show different signal changing rates.

We design a family of feasible spatial transformations $T_{k}\left(\cdot \right)$. Each transformation $T_{k}\left(O_{j}\right)$ is an atomic operation that fine-tunes the location and shape of a trace $O_{j}$, which may also affect the quality of all the corresponding maps. A multi-dimensional data alignment solution is a sequence of transformations T applied to the collected traces. For a solution T, the quality of $M_{i}$ is $Q(M_{i}|T)$. Then, the multi-dimensional data alignment problem can be expressed as follows:

(2) $$arg\max_{T}\;\sum_{i}Q(M_{i}|T).$$

In the following subsections, we will firstly present our basic fusion algorithm and then discuss some design issues and data structures in the proposed approach.

### 4.2. Basic Fusion Scheme

As we have discussed above, the crowdsourced traces are noisy and distorted. To address this problem, we propose a novel scheme that aims to find an optimal transformation T to align the collected data traces and thus maximizes the resulting *ambient maps’* quality.

Before introducing the detailed algorithm design, we firstly discuss how to assess the quality of an *ambient map*. The *ambient map* in a specific resolution is generated by fusing the observed values in a small region or called cells from all the data traces.

Note that the physical parameters exhibit unique features in their value distributions. If the collected data traces are well aligned with the accurate locations, all of the crowdsourced traces will follow the distribution of the physical parameter in each dimension, and thus different traces will be consistent with each other. As the ground truth of the underlying physical value is unknown in common crowdsourced applications, we assess the quality of *ambient maps* through measuring the data consistency among different traces. Then, for each pair of traces, we find their overlapped segments and calculate the correlation score between them. Because the crowdsourced data are noisy due to the device variation, during the computation of the correlation score between trace segments, we focus on the variation trend of the data series instead of the values in each single cell. In this approach, we apply the *Pearson correlation coefficients* to measure the correlation of two series. For each dimension, we have:

(3) $$\begin{array}{c} r_{uv}^{i}=\frac{\sum_{j=1}^{N}\left(u_{j}-\bar{u}\right)\left(v_{j}-\bar{v}\right)}{\sqrt{\sum_{j=1}^{N}{\left(u_{j}-\bar{u}\right)}^{2}}\sqrt{\sum_{j=1}^{N}{\left(v_{j}-\bar{v}\right)}^{2}}}. \end{array}$$

In the equation, $u_{j}$ and $v_{j}$ are the values of series *u* and *v* on the *j*th overlapped location. $r_{uv}^{i}$ is their correlation of the *i*th dimension. Then, the final correlation score is the summation of $r_{uv}^{i}$ from all dimensions. We further extend the correlation computation to the multi-layer case, in which the ambient data map is represented in different dimensions. The details of the multi-resolution ambient map will be discussed in the following subsection. The quality of an *ambient map* is assessed by the correlation scores of all the overlapped trace segments:

(4) $$\begin{array}{c} R=\sum_{u\neq v}R_{uv}=\sum_{u\neq v}\sum_{i}r_{uv}^{i}\times l, \end{array}$$

where $R_{uv}$ is the all dimensional correlation of series *u* and *v*, and *l* is the length of the overlapped series.

In the fusion process, we move and stretch some traces, and thus make the highly correlated ambient data series be located along the same path to maximize the overall correlation score. The transformation of each trace may bring some new overlapped segments, which may contribute new correlation scores and destroy some existing overlapped segments as well. Thus, the effect of each transformation can be represented by a correlation gain function $g(T\left(O_{j}\right))$ that is defined as the difference of the *map correlation score* before and after the trace transformation as follows:

(5) $$\begin{array}{c} g\left(T\left(O_{j}\right)\right)=R^{{}^{\prime }}-R. \end{array}$$

In each iteration, we choose a transformation with a maximum gain and then update the correlation score of the integrated *ambient map* *R*.

The number of potential transformations increases exponentially with the number of traces. In order to speedup the optimization process, we present several pruning strategies. Firstly, the traces usually start from some specific locations, for example, the entrance of the building. Since the location errors will accumulate as the trace length increases, the trace segments near the entrance can be more accurate and easier to align. Thus, we conduct an ordered search starting from such entrance points. In the first step, we only consider traces near the building entrance. Secondly, we consider some restrictions of the transformation. If the indoor maps are available, the transformations are guided by the structures of the indoor map. In most cases, it is reasonable to assume that the trace distortion can be bounded by some thresholds (as the traces can be not very long), thus we let the size of each transformation be less than certain thresholds. Thirdly, in order to further reduce the solution space, we propose to extract transformations with high potential gain. For each pair of traces, we calculate the possible highest correlation score between them after some transformation. That is, we slip one trace from the beginning of the other to the end, during which we find the overlapped segments with the highest correlation score. Then, the transformation that can align the overlapped segments to be physically coincided will be regarded as a good candidate.

### 4.3. Multi-Resolution Ambient Map

As mentioned above, we introduce the concept *ambient map* to model the multi-dimensional ambient data distribution.

**Figure 3:**
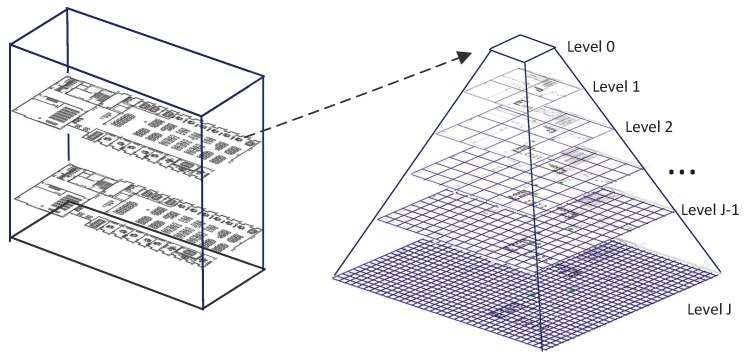
Illustration of the multi-resolution building map.

In this paper, we introduce a multi-resolution pyramid grid structure to encode the indoor maps as shown in Figure 3. Then, an ambient map is the combination of multiple weighted data layers in different resolutions. Different dimensions of data may show different changing rates and thus their significance on different resolutions are different. For example, the indoor temperature changes slowly over spatial locations, so we assign relatively low weight to the high resolution layer of temperature map because of the fact that they contribute little information in the trace alignment. In contrast, the Wi-Fi signal strength dimension is more location-sensitive, and we will lose valuable information if this signal is mapped to a low-resolution map.

Formally, the indoor map of the building is represented by grids of different scale. The map for a building contains a tree of floors for the whole building and a quadtree for each floor. Moving up the pyramid, both size and resolution decrease. For level *k*, we set its size as $2^{k}\times 2^{k}$. Then, the map for dimension $m_{i}$ of a building with the pyramid description is $M_{i}^{k}\left(v,w,f,t\right)$, here *k* is its resolution level, (v,w) is the location of the grid, and *f* indicates the floor level.

### 4.4. Trace Coding

**Figure 4:**
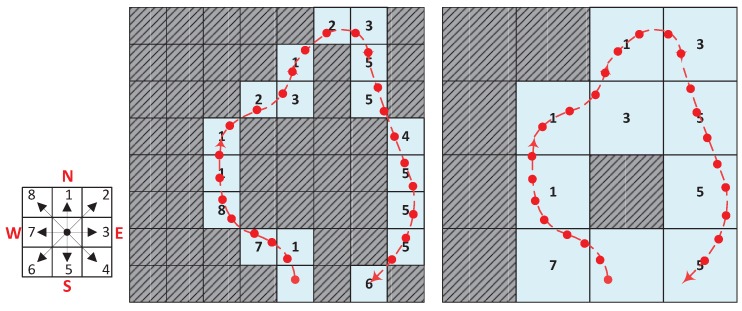
Trace coding projects a raw moving trace to a grid-based 8-directional chain coded presentation.

For a continuous trace $O=\{\left(o_{j},t_{j},p_{j}\right)\}$, the location $p_{j}=\left(x_{j},y_{j},z_{j}\right)$ in the earth coordinate can be projected to the grid representation $\left(v_{j},w_{j},f_{j}\right)$ by the nearest neighbor rule as shown in Figure 4, e.g., if $p_{j}$ is in the range of a grid, then it is projected to this grid. Note that the absolute location $p_{j}$ of the crowdsourced data traces cannot be accurately determined in many indoor situations. Nevertheless, the shape of traces can be estimated using on-board sensors, like accelerometer and gyroscope, which provide information about the user’s movements and thus help us estimate the shape of trace segments. In order to utilize such kind of raw traces without absolute locations, as well as facilitate the trace representation, storage and optimization process, we propose an 8-directional chain code to model a crowdsourced data trace. As mentioned above, we apply multi-resolution grids to divide the physical map. In this case, we can assign the start point $p_{0}$ of a trace to a start cell *S*, and the consequent points are projected into a series of cells, thus the whole trace can be embedded into the grid. For each pair of successive cells, we define *c* as the moving direction from the start cell to the end one. In this work, direction *c* is coded using a 3-bit code. Then, the locations of an integrated trace can be represented by a start cell and a sequence of direction codes. The observed data of each point will be associated with corresponding cells. For each cell, the observed data of dimension $m_{i}$ is the mean value of all readings located in this cell. A coded trace is $CO=\{P_{s},\left\{o_{j},t_{j},c_{j}\right\}\}$, here $P_{s}$ is the start cell and *c* is a 3-bits direction. In the implementation, the coded trace of the supported highest resolution will be constructed firstly, then the lower resolution trace can be generated from the higher resolution trace. Figure 4 illustrates the direction codes and examples of coded trace in different resolutions. The direction codes of the trace in a lower level (higher resolution) is “17811231235545556”, and in a higher level is “711313555”.

In cases that the absolute coordinates of some points in a trace can be obtained at GPS capable positions, e.g., the entry of a building or the balcony, we treat these GPS-enabled points as anchors for the trace. We call the trace with anchors a *rooted trace*. In this work, we focus on the rooted trace with only a fixed start point, which is the most common situation because it is easy to obtain the GPS location of the building entrance. The traces with anchors at the end point can be simply reversed to traces with anchors at the start point. For those traces with more than one anchor, the segments between two anchors can be easily aligned, leaving the rest of the segments with only one anchor fall into the rooted trace situation. Therefore, we define the rooted traces as the following statement:

**Definition** **1** (Rooted Trace). *The rooted trace contains one anchor at the start point, whose accurate location is known, i.e., $P_{s}$ is known for trace $CO=\{P_{s},\left\{o_{j},t_{j},c_{j}\right\}\}$.*

Sometimes, a user may turn on the tracking service after he/she enters the building, and the trace has no anchor point, i.e., only direction codes are available, We call this kind of trace the floating trace.

**Definition** **2** (Floating Trace). *The floating trace doesn’t have any anchor point, i.e., $P_{s}$ is unknown for trace $CO=\{P_{s},\left\{o_{j},t_{j},c_{j}\right\}\}$.*

As we can see, giving the start point of one floating trace makes it become a rooted trace. In our method, the rooted traces are processed first, and then the floating traces will be further considered.

### 4.5. Trace Transformation

**Figure 5:**
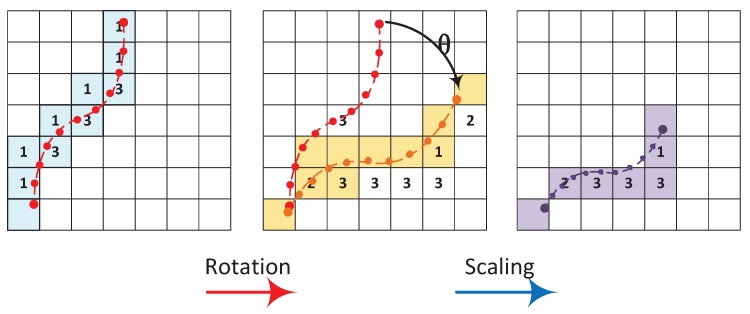
Illustration of different trace transformation operations.

To align the trace for data fusion, a sequence of transformations will be applied to the trace $\left(x^{\prime },y^{\prime }\right)=T\left(\left(x,y\right)\right)$. In this paper, we employ the affine transformation, as shown in Figure 5, which has the general form of

(6) $$\begin{array}{c} \left[x^{\prime }\;y^{\prime }\;1\right]=\left[x\;y\;1\right]\times T=\left[x\;y\;1\right]\times \left[\begin{array}{ccc} t_{11} & t_{12} & 0\\ t_{21} & t_{22} & 0\\ t_{32} & t_{32} & 1 \end{array}\right]. \end{array}$$

Considering the main misalignment caused by the distance and orientation miscalculation while the user moves in the building, the rotation and scaling transformations are defined for the rooted trace and one additional translation transformation for the floating trace.

1. Rotation:
  (7) $$\begin{array}{c} T_{r}=\left[\begin{array}{ccc} \cos\theta & \sin\theta & 0\\ -\sin\theta & \cos\theta & 0\\ 0 & 0 & 1 \end{array}\right]. \end{array}$$
2. Scaling:
  (8) $$\begin{array}{c} T_{s}=\left[\begin{array}{ccc} c_{x} & 0 & 0\\ 0 & c_{y} & 0\\ 0 & 0 & 1 \end{array}\right]. \end{array}$$
3. Translation:
  (9) $$\begin{array}{c} T_{t}=\left[\begin{array}{ccc} 1 & 0 & 0\\ 0 & 1 & 0\\ t_{x} & t_{y} & 1 \end{array}\right]. \end{array}$$
  We can obtain:
  (10) $$\begin{array}{c} T=T_{r}\cdot T_{s}\cdot T_{t}=\left[\begin{array}{ccc} c_{x}\cdot \cos\theta & c_{y}\cdot \sin\theta & 0\\ -c_{x}\cdot \sin\theta & c_{y}\cdot \cos\theta & 0\\ t_{x} & t_{y} & 1 \end{array}\right]. \end{array}$$

It shall be noted that $c_{x}$ and $c_{y}$ can be treated as equal for the trajectory of dead reckoning in the scaling operation, thus it can be regarded as a similarity transformation. The reason we choose similarity transformation rather than affine transformation is that the trajectory of dead reckoning does not need shearing and flipping, which is a difference between similarity transformation and affine transformation. The other reason is that the similarity transformation has four degrees of freedom, while the affine transformation has six. Thus, the problem scale can be reduced. The trace coding discretizes the trace presentation and simplifies the transformation, since the rotation and scaling operations are only applied to the direction codes of a trace, despite its start point. In addition, we use nearest neighbor interpolation to generate the trace after transformation. The translation is even simpler by just changing the start point $P_{s}$, leaving the rest of the trace unchanged.

We observe from our collected trace data that, in most cases, the deviation of trace directions is within 20 degrees, and the offsets of the coordinates are within 3 m, so we restrict the size of transformations to each trace. In addition, to avoid an infinite search space, we assign discrete values to the three transformation operations. For example, we select eight rotation angles between anticlockwise 20 degrees and clockwise 20 degrees. The scaling of traces is set to 10 different levels from 2% to 10% expansion or reduction.

Another design issue is the granularity of the trace transformation. Integrated data traces sometimes exhibit long length and complex formation. With the three types of transformation operations, it is efficient to adjust traces with a relatively simple structure like a straight path. It, however, is difficult to fine-tune some local formations in a complex trace. To address this issue, we propose to segment the data traces and conduct transformations one by one. Each entry in a coded trace denotes the direction of the next cell, so the difference between consecutive items indicates angle of the direction change of the trace. One unit difference of the code numbers corresponds to the path direction change of 45 degrees and, in this work, we define the direction change equal to or more than 90 degrees (two unit difference) as a significant change of the path way. Then, we divide the trace according to positions that experience significant direction changes.

Defining these transformations, our fusion algorithm also needs anther preparation. Because of a variety of localization methods, the noises and distortions of crowdsourced traces are of great complexity. In order to align the traces and recover their real shapes, we need to separate the traces into segments according to the shapes of traces, and transform all the segments to find the optimal transformations. Intuitively, with our trace coding method, we only need to check each of the coded traces, and cut it when the its code number changes. However, the fact is more complex. The code of the first trace shown in Figure 5 is “113131311”. If we segment this trace by the code number, it should be cut into seven segments, most of which are only one grid length, so that they cannot be transformed. In fact, in this case, cutting into two segments should be appropriate. As trace coding eliminates many direction features of the traces, we prefer to do segmentation on the non-code traces. For the raw traces, we can use Biezer curve least square fitting [39] to do the segmentation. Then, we code all segments as described before. Of course, during this process, though being transformed, the segments of a trace should keep being linked together.

## 5. Experiments and Evaluation

### 5.1. Experiment Setup

**Figure 6:**
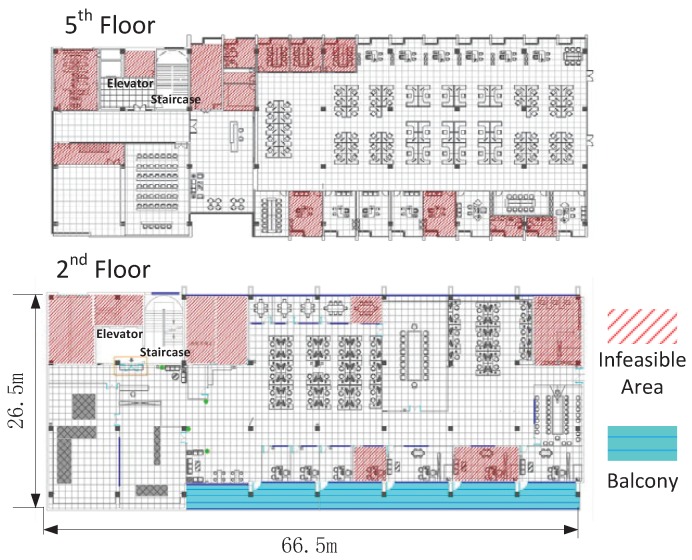
The ground truth of the floor plan for the 5th floor and the 2nd floor.

We implement a crowdsourcing data collection application in five different types of commodity smart phones, including SamSung e120s, SamSung e120k, HTC one 3D, HTC one X and HTC one. We conduct the indoor data trace in a large lab building. The data traces are mainly collected on two floors with a total floor space of 4000 m${}^{2}$. Our data traces span different indoor infrastructures, such as the corridor, working place, exhibition hall, office, meeting room, and the like. Figure 6 illustrates the floor plan of these two floors.

In total, 26 volunteers with heights from 160 cm to 185 cm join this experiment, who carry smart phones of different models with our indoor trace collection application during their daily work. The data collection lasts for 12 days and we get 931 traces in all. Three kinds of ambient information are collected, including the signal strength of all sensible Wi-Fi APs, the signal strength of the phone base station and the magnetic field. There are 11 APs deployed on these two floors.

### 5.2. Evaluation

In order to test and evaluate our data fusion method, we need a method to know the traces of the crowdsourced data to simulate the distorted results of the inaccurate indoor localization services. We describe the design and implementation of our crowdsourcing approach to obtain indoor multi-dimensional traces without any pre-knowledge of the building using the on-board sensors in off-the-shelf smart phones. The captured acceleration values are in the coordinate system fixed to the smart phone, and here we refer it as a phone coordinate system. Considering a user could hold the phone in any position, we firstly convert the acceleration from the phone coordinate system to the earth coordinate system, i.e., north, east, gravity.

With the on-board accelerometer of a smart phone, a user’s movement can be sensed and used for activity recognition. In our work, we recognize the following most common activities: walking, climbing stairs and taking an elevator. We use these events, climbing stairs and taking an elevator, to decide the beginning of detecting walking trace. Knowing the beginning of walking, we need a detecting algorithm to detect the walking trace by using only smart phones. We implement the dead reckoning method described in Montage [40] on both Android and IOS smart phones to simulate localization services.

**Figure 7:**
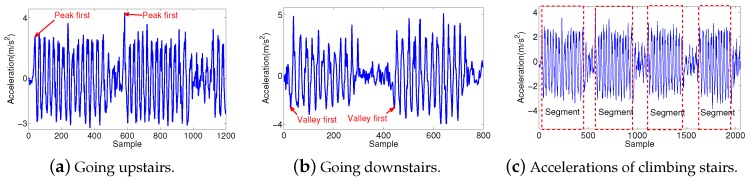
Accelerations of climbing stairs.

The method we recognize for the three activities is based on the following observation. As shown in Figure 7, accelerations generated by common walking changes positively and negatively regularly. Each step consists of a continuous sequence of positive accelerations and a followed sequence of negative accelerations that have similar amplitudes. The recognizing step method is included by the trace detecting algorithm mentioned before. In fact, climbing stairs is a kind of walking, and its acceleration springs profile is similar to that of walking, as shown in Figure 7. As presented in Figure 7a,b, the acceleration springs’ profiles of climbing upstairs and going downstairs are very similar. Their main difference is climbing upstairs starting with a peak and going downstairs beginning with a valley. As shown in Figure 7c, according to the changing amplitude levels, the acceleration data can be separated into several segments, in which the segments have higher amplitude level corresponding to steps of climbing processes, and the lower amplitude parts stand for walking on platforms.

**Figure 8:**
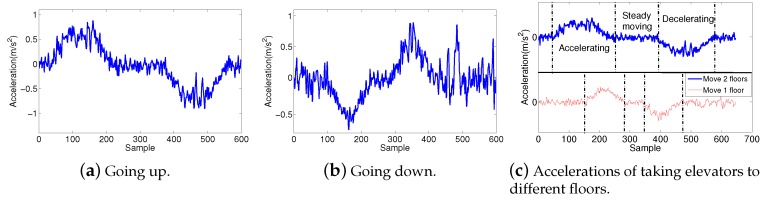
Accelerations of taking elevators.

Figure 8 demonstrates the acceleration profiles of taking an elevator. As shown in Figure 8a, when taking an elevator upstairs, we find that the trend of acceleration increases significantly at the beginning, then it returns to nearly zero and lasts a few samples; finally, it decreases and returns to about zero. The amplitude and duration time is quite similar between the increase–return process and the decrease–return process. Taking an elevator downstairs is the opposite of the upstairs process, as shown in Figure 8b. The profiles of taking an elevator to different floors are very similar. The only difference is the duration of the steady moving process, as shown in Figure 8c. These significantly distinguishing patterns can be distinguished by a simple Deterministic Finite Automaton (DFA) based algorithm: at the beginning, we use a low-pass filter to extract the acceleration whose spectrum is lower than 5 Hz. Then, use the step detecting algorithm in Montage [40] to detect and count steps. If we find that the difference between average amplitude of 8–15 successive steps and the following 2–5 steps is more than 1, we suppose that the user is climbing stairs. In addition, if one cannot detect any steps, we begin to detect the elevator patterns. Specifically, build a simple DFA to find the three segments of the acceleration, and compare the durations and maximum amplitudes of the first and the third segment; if their differences are lower than 5%, and the standard deviation of the second segment is lower than 0.3 and the average value is lower than 0.2, we suppose the user is taking an elevator.

**Figure 9:**
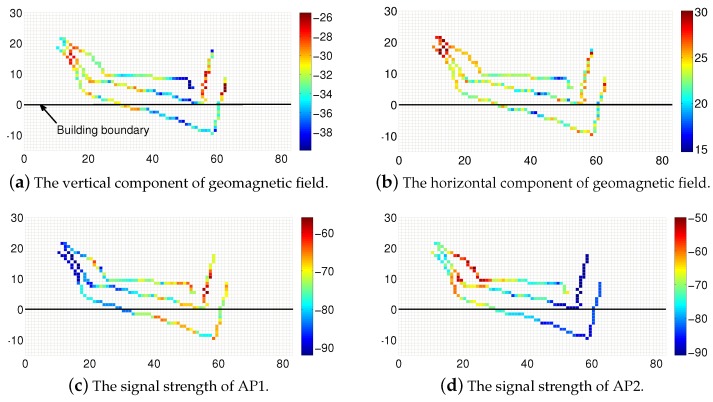
The ambient data map on different dimensions.

Figure 9 shows different dimensions of ambient data from the users, including the vertical component of the geomagnetic field, the horizontal component of the geomagnetic field, signal strength of AP1, and signal strength of AP2. The color of each cell indicates the ambient value of each cell, and we can clearly find the data distribution of each dimension. In this experiment, we examine the features of the collected multi-dimensional traces. With the on-board accelerometer and gyroscope sensor readings, we utilize the dead reckoning method [40] to track the user’s movement. Three pieces of data traces are collected from three different users. For these three users, they start from the same point, that is, the entrance of the floor, and share a common pathway. Then, they walk to their own seats.

We can see that the traces experience significant distortion on their locations. The recorded coordinates of some points are outside of the building boundary. In addition, it is easy to find that three traces exhibit nearly the same data change trend on the first half of the traces. Similar trends can be observed in the other three dimensions as well. It seems very likely that the three users share a common path on the first half of the trace that meets the ground truth. Thus, we are confident enough to conduct transformation on the three traces and move them together in the first half. In fact, it is hard to determine the concrete transformations just based on the information in the snapshot. In the integrated optimization, we need more information to make decisions such as the moving direction, scaling size, and the like.

**Figure 10:**
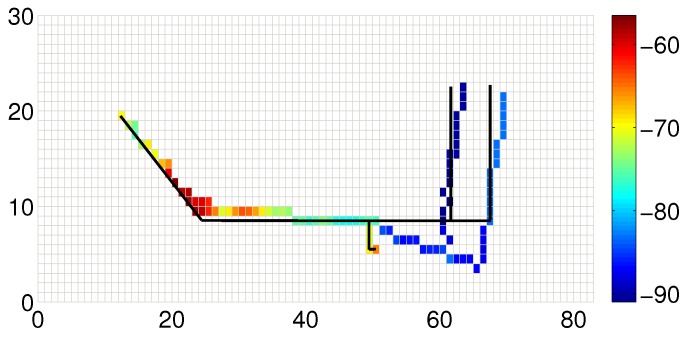
An example of the trace alignment result.

**Figure 11:**
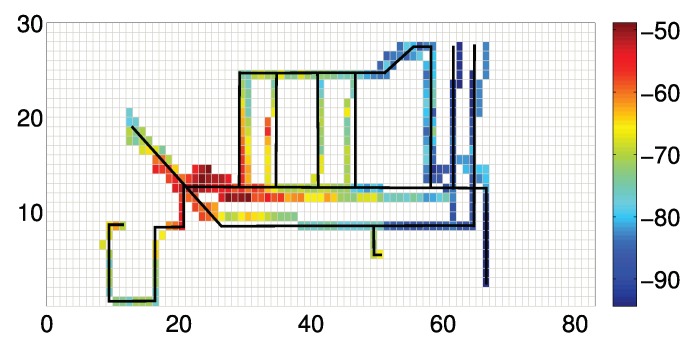
Map reconstruction with the aligned data traces.

Figure 10 shows the fusion results of the traces in Figure 9. The solid black line denotes the real route of three users. The optimized traces match the groudtruth, especially in the first half part. The latter half, however, still deviates from the real path. Through careful examination of the trace data, we find that the result is due to the lack of enough information for generating the optimization decision. It shall be noted that the accumulative error may deteriorate the dead reckoning performance, especially when the trajectory is long and has many turns. Although the end point of the trajectory has huge offset, each short segment (for indoor cases, the segments are usually not long) is not very erroneous (despite the initial offset). Otherwise, after segmentation, each segment’s shape is always quite similar to the ground truth. Thus, we can use a ‘small’ similarity transformation to cancel the error. Actually, the length of the traces will not obviously affect the accuracy, except when all the paths are very short. In Figure 11, we show an integrated data map constructed by 11 different users after data alignment. We can find that the combined traces precisely sketch the structure of the underlying indoor map.

**Figure 12:**
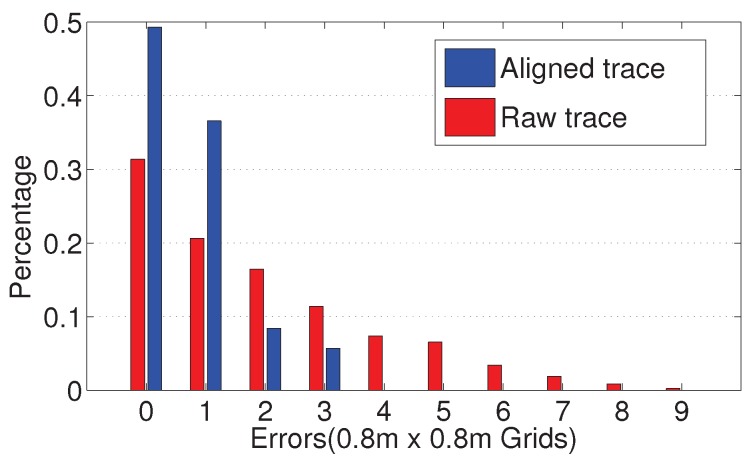
Location deviation of crowdsourced traces.

Finally, we summarize the location deviation in traces before and after the optimization, and the results are illustrated in Figure 12. In this experiment, we choose a 0.8 m × 0.8 m grid as the distance unit that equals the size of the floor tile. Nearly 50% of the trace points are aligned to their right locations and around 90% of points show deviations within two cells. Compared with the unaligned traces, the optimized traces achieve significant improvement on the accuracy.

## 6. Conclusions

In this work, we extensively studied the multi-dimensional crowdsourced data fusion problem and proposed a novel method to fuse the noisy and distorted crowdsourced data from different types of sensory data together in different situations. We proposed a new trace coding and transformation approach to conduct the trace alignment, in order to deal with complex trace distortion issues. Moreover, we proposed an effective fusion scheme to find the crowdsourced trace alignment solution. Finally, we implemented the proposed approaches on different commercial devices and conducted experiments to verify the effectiveness of our method. Since our system is an initial and general framework, and the similar works are limited so far, we did not compare it with the dedicated systems in this paper. In our future work, we will spend more time to make a comparison to some similar works to demonstrate our merits and limitations, and improve our current work.
